# Randomized controlled trial of the effects of consumption of ‘Yabukita’ or ‘Benifuuki’ encapsulated tea-powder on low-density lipoprotein cholesterol level and body weight

**DOI:** 10.1080/16546628.2017.1334484

**Published:** 2017-06-19

**Authors:** Yuko Igarashi, Taku Obara, Mami Ishikuro, Hiroko Matsubara, Michiko Shigihara, Hirohito Metoki, Masahiro Kikuya, Yoichi Sameshima, Hirofumi Tachibana, Mari Maeda-Yamamoto, Shinichi Kuriyama

**Affiliations:** ^a^ Department of Molecular Epidemiology, Tohoku University Graduate School of Medicine, Sendai, Japan; ^b^ Tohoku Medical Megabank Organization, Tohoku University, Sendai, Japan; ^c^ Department of Pharmaceutical Sciences, Tohoku University Hospital, Sendai, Japan; ^d^ International Research Institute of Disaster Science (IRIDeS), Tohoku University, Sendai, Japan; ^e^ Department of Internal Medicine, Omaezaki Municipal Hospital, Omaezaki, Japan; ^f^ Department of Bioscience and Biotechnology, Faculty of Agriculture, Kyushu University, Fukuoka, Japan; ^g^ Food Research Institute, National Agriculture and Food Research Organization, Ibaraki, Japan

**Keywords:** Green tea, Benifuuki green tea, cardiovascular disease, O-methylated epigallocatechin gallate, dyslipidemia

## Abstract

**Background**: Previous studies have reported controversial results for the association between green tea consumption and low-density lipoprotein (LDL)-cholesterol and body weight.

**Objective**: The objective of this trial was to determine the effects of two kinds of green tea on LDL-cholesterol and body weight.

**Methods**: We randomly assigned 151 participants (98 men, 53 women) aged 30–70 years into three groups: Yabukita green tea group, Benifuuki green tea group, or placebo group. Participants consumed 1.8 g/day of green tea extract powder or placebo for 12 weeks. The primary outcomes were LDL-cholesterol level and body weight, and the secondary outcomes were risk factors for cardiovascular disease.

**Results**: Both Yabukita and Benifuuki green tea significantly lowered LDL-cholesterol. The magnitudes of the lipid-lowering effect of both types of tea were significantly larger than that of placebo. No differences with respect to changes in LDL-cholesterol were observed between the Yabukita and Benifuuki green tea groups. Neither Yabukita nor Benifuuki green tea had any effect on body weight and no difference was observed among groups regarding changes in body weight.

**Conclusion**: Both Yabukita and Benifuuki green tea lowered LDL-cholesterol, and the lipid-lowering effects of these two green teas were not different. Neither tea lowered body weight.

## Introduction

Associations between green tea consumption and cardiovascular disease (CVD) have been reported by many epidemiological studies [1]. Participants consuming at least five cups of green tea daily had significantly lower overall mortality, CVD mortality, and stroke mortality than those consuming less than one cup daily, in the Ohsaki National Health Insurance Cohort Study, a longitudinal study that followed 50,000 people for 11 years [2]. In a meta-analysis of 18 prospective cohort studies regarding the association between tea consumption and mortality, summary relative risks for the highest versus lowest category of green tea consumption were 0.67 [95% confidence interval (CI) 0.46, 0.96] for CVD mortality and 0.80 (95% CI 0.68, 0.93) for all-cause mortality, and the dose–response analysis indicated that a one-cup dose increment of green tea consumption was associated with a 5% lower risk of CVD mortality and a 4% lower risk of all-cause mortality [3].

Green tea contains various antioxidative flavan-3-ols (tea catechins), such as (–)-epigallocatechin gallate (EGCG), which exerts potent inhibitory effects on low-density lipoprotein (LDL) oxidation *in vitro* and *ex vivo* in humans [4]. EGCG is the most prevalent catechin in green tea and it decreases the micellar solubility of cholesterol via specific interaction with phosphatidylcholine [5,6]. Previous studies have found that continuous intake of green tea containing catechins and caffeine (five or more cups per day) may be beneficial for body weight management, vascular disease risk reduction via LDL-cholesterol improvement, and type 2 diabetes risk reduction through the lowering of fasting blood glucose levels [7]. In a randomized double-blind placebo-controlled study using a beverage containing 392.2 mg of tea catechins with a galloyl moiety, Suzuki et al. reported that visceral fat area was significantly reduced in the catechin group compared with the placebo group after 12 weeks of daily consumption [8]. In another randomized double-blind placebo-controlled study using beverages containing 41.1 mg, 444.3 mg, or 665.9 mg of tea catechins with a galloyl moiety, Kajimoto et al. reported that visceral fat area, total cholesterol, and LDL-cholesterol significantly decreased in all catechin groups compared with the control group [9]. However, epidemiological evidence of the cardiovascular effects of diets rich in flavonoids, including catechins, is controversial [1,^10^].

Epigallocatechin-3-*O*-(3-*O*-methyl)-gallate (EGCG3”Me) is thought to be a major effective compound of catechins. EGCG3”Me is contained in Benifuuki green tea but not in Yabukita green tea [11]. A previous study reported that tea containing unique methylated catechins such as EGCG3”Me significantly down-regulated the hepatic expression of lipid-synthesis genes such as sterol regulatory element-binding protein *c*, acetyl-coenzyme A (CoA) carboxylase, fatty acid synthase, and stearoyl-CoA desaturase-1 [12]. To our knowledge, there has only been one randomized controlled trial (RCT) that focused on the effects of different kinds of green tea [13].

The objective of this RCT was to determine the effects of two kinds of green tea on LDL-cholesterol and body weight.

## Methods

### Design

This was a double-blind, placebo-controlled, three-parallel intergroup trial. We randomly assigned 151 participants (98 men and 53 women) aged 30–70 years to one of three groups: the Yabukita green tea group, the Benifuuki green tea group, or the placebo group. Participants consumed 1.8 g/day of green tea extract powder or placebo for 12 weeks.

### Study participants

The participants in the present study were recruited using newspapers and flyers. The inclusion criteria for the intervention program were: (i) both sexes; (ii) age between 30 and 70 years; (iii) body mass index (BMI) more than 23 kg/m^2^; and (iv) total cholesterol more than 200 mg/dl or LDL-cholesterol more than 120 mg/dl. The exclusion criteria were: (i) taking medication for dyslipidemia, hypertension, or diabetes mellitus; (ii) history of ischemic heart disease or cardiac insufficiency; (iii) pharmaceutical product or food allergy; (iv) pregnant or lactating women; and (v) persons otherwise deemed inappropriate for this study. Eligible participants were stratified by sex (men or women), age (in years), and individual baseline variables, and randomization was conducted using the permuted block method with a two from four-person block. Participants’ data were collected at the Tokuiku Health Promotion Center and the Daitou Health Promotion Center in Kakegawa, Japan.

**Figure 1.**
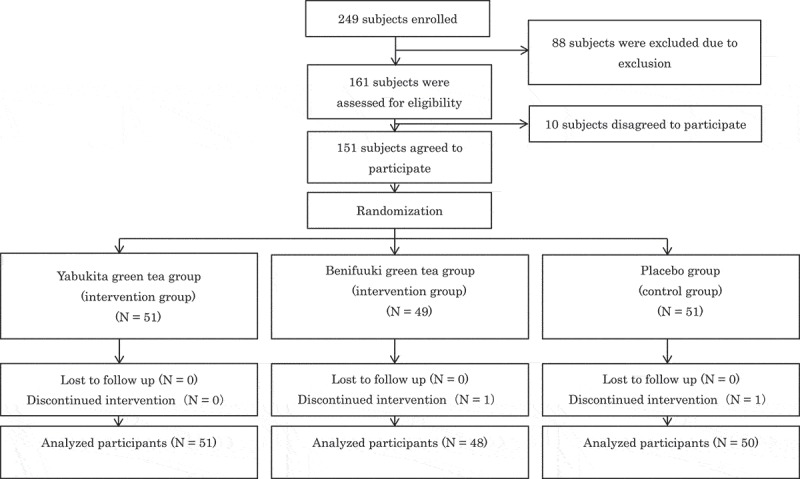
Trial flowchart. We asked 249 individuals to participate in this study and obtained informed consent from 151 individuals. These 151 participants were randomly assigned by an epidemiologist to the Yabukita green tea group (*n* = 51), the Benifuuki green tea group (*n* = 49), or the placebo group (*n* = 51).

We used 3 ± 5 mg/dl to determine a detectable difference in LDL-cholesterol, that is smaller value compared to the magnitude of reduction in LDL-cholesterol (5.30 mg/dl) by green tea consumption as in a previous meta-analysis [13], when comparing the LDL-cholesterol-lowering effect between the two kinds of green tea. Therefore, a minimum sample size of 44 participants per group was required to detect a difference (power = 80%, two-sided alpha = 0.05). We asked 249 participants to participate in this study and obtained informed consent from 151 participants. These 151 participants were randomly assigned by an epidemiologist to the Yabukita green tea group (*n* = 51), the Benifuuki green tea group (*n* = 49), or the placebo group (*n* = 51) (Figure 1).

The participants were asked to take 10 capsules a day that contained either Yabukita or Benifuuki green tea extract powder or placebo. Each capsule contained 180 mg of tea extract powder or starch (placebo). Participants were asked to take three to four capsules after each meal during the intervention period and were asked not to drink any other catechin-containing beverages, but other beverages and supplements were allowed.

The study participants were asked to stop consuming catechin-containing beverages 2 weeks before beginning the study (washout period), following which they were to start consuming the provided green tea capsules every day for 12 weeks. The study was conducted between September 2009 and December 2009. The first assessments were conducted from 12 September 2009 to 19 September 2009. Intermediate assessments were conducted from 24 October 2009 to 31 October 2009. The final assessments were conducted from 5 December 2009 to 19 December 2009. The primary outcomes were LDL-cholesterol and body weight. The secondary outcomes were waist circumference, total cholesterol, high-density lipoprotein (HDL)-cholesterol, triglycerides, fasting plasma glucose, glycosylated hemoglobin (HbA1c), insulin, serum amyloid A, high-sensitivity C-reactive protein (hsCRP), serum adiponectin, and 8-hydroxy-2’-deoxyguanosine (8-OHdG; a major form of DNA damage induced by reactive oxygen species, which is receiving increasing attention).

### Statistical analysis

Comparisons among the three groups to assess differences in biochemical and anthropometric parameters at baseline were performed by analysis of variance (ANOVA). The sex ratio was compared by a chi-squared test. The effects of the intervention on LDL-cholesterol level, body weight and other outcome measures were tested using a paired *t* test in each group before and after the intervention. Analysis of covariance was also used to investigate the net changes among the three groups. In the analysis of net changes, the following variables were considered potential confounders: age at baseline in years (continuous variable), sex, and baseline level of each variable. All statistical analyses were performed using SAS version 9.4 (SAS, Cary, NC, USA). Intention-to-treat analysis was adopted. Approximate variance formulae were used to calculate the 95% CIs. Differences were accepted as statistically significant at *p* < 0.05.

The study protocol was reviewed and approved by the Ethics Committee of Tohoku University Graduate School of Medicine, and registered at clinicaltrials.gov (trial number UMIN000003314).

## Results

All study participants were successfully followed up and no apparent harmful effects were observed. Comparisons of baseline variables between the Yabukita green tea group, the Benifuuki green tea group, and the placebo group are shown in Table 1. No significant differences in baseline LDL-cholesterol level and body weight were observed among the three groups.

**Table 1.** Baseline characteristics of participants in the intervention groups (Yabukita and Benifuuki green tea groups) and control group (placebo).

Variable	Yabukita group (*n* = 51)	Benifuuki group (*n* = 49)	Placebo group (*n* = 51)	*p*^a^
Age (years)	47.2 ± 9.7	47.2 ± 9.0	47.9 ± 9.7	0.93
Women (%)	35.3	36.7	33.3	0.94
Body weight (kg)	67.2 ± 13.7	67.3 ± 15.4	70.1 ± 12.5	0.48
Body mass index (kg/m^2^)	24.3 ± 3.2	24.6 ± 4.0	25.6 ± 3.3	0.14
Waist circumference (cm)	87.4 ± 8.6	86.7 ± 10.4	88.1 ± 7.6	0.74
Total cholesterol (mg/dl)	217.1 ± 30.3	220.5 ± 28.2	207.9 ± 32.2	0.10
LDL-cholesterol (mg/dl)	146.3 ± 29.1	149.5 ± 26.2	142.2 ± 29.5	0.44
HDL-cholesterol (mg/dl)	56.0 ± 13.8	57.9 ± 13.6	53.6 ± 11.0	0.26
Triglycerides (mg/dl)	132.5 ± 86.1	111.3 ± 46.8	122.2 ± 63.5	0.30
Serum adiponectin (μg/ml)	10.5 ± 4.6	10.5 ± 4.5	9.8 ± 4.7	0.67
Fasting plasma glucose (mg/dl)	87.7 ± 11.1	86.9 ± 8.5	90.0 ± 15.5	0.41
hsCRP (mg/dl)	96.4 ± 152.7	185.5 ± 289.4	111.1 ± 175.5	0.09
HbA1c(%)	5.1 ± 0.3	5.1 ± 0.2	5.2 ± 0.4	0.36
Insulin (μU/ml)	9.1 ± 2.1	8.6 ± 2.1	10.6 ± 7.4	0.08
Serum amyloid A (μg/ml)	8.5 ± 3.2	14.7 ± 24.2	16.8 ± 55.5	0.47
8-OHdG concentration (ng/ml)	12 ± 7.1	11.3 ± 7.9	12.5 ± 7.7	0.75
8-OHdG/CRE (ng/mg creatinine)	9.6 ± 3.7	8.8 ± 3.5	9.2 ± 3.2	0.51
8-OHdG generation rate (ng/kg/h)	8.2 ± 4.1	7.7 ± 5.4	7.6 ± 4.1	0.80

Data are shown as mean ± SD.

^a^Chi-squared test for female ratio and anthropometric parameters, and analysis of variance for biochemical parameters.

LDL, low-density lipoprotein; HDL, high-density lipoprotein; hsCRP, high-sensitivity C-reactive protein; HbA1c, glycosylated hemoglobin; 8-OHdG, 8-hydroxy-2’-deoxyguanosine; CRE, creatinine.

Table 2 shows changes in LDL-cholesterol levels and body weight. After 12 weeks of green tea consumption, the mean ± SD changes from baseline in LDL-cholesterol level were −7.7 ± 15.8 mg/dl in the Yabukita green tea group, −10.7 ± 21.1 mg/dl in the Benifuuki green tea group, and 0.04 ± 13.2 mg/dl in the placebo group. We found a significant difference between the Yabukita green tea group and the placebo group with respect to changes in LDL-cholesterol (−6.9 mg/dl; 95% CI −13.5, −0.3 mg/dl). The difference between the Benifuuki green tea group and the placebo group was significant at −9.4 mg/dl (95% CI −16.1, −2.7 mg/dl), but no significant difference was found between the Yabukita green tea group and the Benifuuki green tea group (−2.5 mg/dl; 95% CI −9.1, 4.2 mg/dl). After 12 weeks of green tea consumption, the mean ± SD changes from baseline in body weight were 0.6 ± 4.2 kg in the Yabukita green tea group, 0.3 ± 1.3 kg in the Benifuuki green tea group, and 0.5 ± 1.2 kg in the placebo group. With respect to changes in body weight, we found no significant difference between the Yabukita green tea group and the placebo group (−0.1 kg; 95% CI −1.1, 0.9 kg), between the Benifuuki green tea group and the placebo group (−0.4 kg; 95% CI −1.5, 0.6 kg), or between the Yabukita green tea group and the Benifuuki green tea group (−0.3 kg; 95% CI −1.4, 0.7 kg).

**Table 2.** Change in body weight and serum lipids of participants in the placebo group, Benifuuki green tea group, and Yabukita green tea group.

Variable	Baseline	After 12 weeks	After 12 weeks minus baseline	*p*^a^	Net change^b^	*p*
LDL-cholesterol (mg/dl)						
Placebo	142.2 ± 29.5	141.7 ± 32.7	0.04 ± 13.2	0.98	−9.4 (−16.1 to −2.7)	0.006 (Benifuuki vs placebo)
Benifuuki	149.5 ± 26.2	138.8 ± 27.6	−10.7 ± 21.1	0.001	−2.5 (−9.1 to 4.2)	0.46 (Benifuuki vs Yabukita)
Yabukita	146.3 ± 29.1	138.2 ± 27.6	−7.7 ± 15.8	0.001	−6.9 (−13.5 to −0.3)	0.04 (Yabukita vs placebo)
Body weight (kg)						
Placebo	70.1 ± 12.5	70.5 ± 12.4	0.5 ± 1.2	0.002	−0.4 (−1.5 to 0.6)	0.43 (Benifuuki vs placebo)
Benifuuki	67.3 ± 15.4	67.6 ± 15.5	0.3 ± 1.3	0.18	−0.3 (−1.4 to 0.7)	0.54 (Benifuuki vs Yabukita)
Yabukita	67.2 ± 13.7	67.6 ± 12.8	0.6 ± 4.2	0.30	−0.1 (−1.1 to 0.9)	0.86 (Yabukita vs placebo)

^a^Baseline values were compared with those obtained after 12 weeks. The significance of the differences was isolated by the paired *t* test.

^b^The change in the Benifuuki or the Yabukita green tea group minus the change in the placebo group; and the change in the Benifuuki group minus the change in the Yabukita group.

Net differences were calculated by analysis of covariance; adjusted for age (years), sex, and individual baseline variables.

LDL, low-density lipoprotein.

There were significant decreases in total cholesterol, Hb_A1c_, insulin, hsCRP, 8-OHdG concentration, and 8-OHdG/CRE and 8-OHdG generation rate in each group, but the net changes between groups were not significant for any of these variables (Table 3). With respect to changes in waist circumference, we found a significant difference between the Yabukita green tea group and the placebo group (−1.5 cm; 95% CI −2.7, −0.3 cm), and between the Yabukita green tea group and the Benifuuki green tea group (1.4 cm; 95% CI 0.1, 2.6 cm). However, no significant difference was observed between the Benifuuki green tea group and the placebo group (−0.1 cm; 95% CI −1.4, 1.1 cm). With respect to changes in HDL-cholesterol, we found a significant difference between the Benifuuki green tea group and the placebo group (2.3 mg/dl 95% CI 0.2, 4.5 mg/dl), and between the Yabukita green tea group and the Benifuuki green tea group (2.7 mg/dl; 95% CI 0.6, 4.8 mg/dl). However, no significant difference was observed between the Yabukita green tea group and the placebo group (−0.4 mg/dl; 95% CI −2.5, 1.7 mg/dl).

**Table 3.** Change in cardiovascular risk factors of participants in the placebo group, Benifuuki green tea group, and Yabukita green tea group.

Variable	Baseline	After 12 weeks	After 12 weeksminus baseline	*p*^a^	Net change^b^	*p*
Waist circumference (cm)						
Placebo	88.1 ± 7.6	87.9 ± 7.9	0.2 ± 2.9	0.58	−0.1 (−1.4 to 1.1)	0.84 (Benifuuki vs placebo)
Benifuuki	86.7 ± 10.4	86.9 ± 9.8	0.1 ± 3.5	0.79	1.4 (0.1 to 2.6)	0.03 (Benifuuki vs Yabukita)
Yabukita	87.4 ± 8.6	86.0 ± 8.6	−1.2 ± 3.0	0.006	−1.5 (−2.7 to −0.3)	0.02 (Yabukita vs placebo)
HDL-cholesterol (mg/dl)						
Placebo	53.6 ± 11.0	55.5 ± 10.6	2.0 ± 5.4	0.01	2.3 (0.2 to 4.5)	0.03 (Benifuuki vs placebo)
Benifuuki	57.9 ± 13.6	61.8 ± 14.4	3.9 ± 5.6	< 0.0001	2.7 (0.6 to 4.8)	0.01 (Benifuuki vs Yabukita)
Yabukita	56.0 ± 13.8	57.1 ± 14.0	1.4 ± 5.2	0.07	−0.4 (−2.5 to 1.7)	0.73 (Yabukita vs placebo)
Total cholesterol (mg/dl)						
Placebo	207.9 ± 32.2	215.2 ± 36.5	7.8 ± 16.0	0.001	−6.6 (−14.9 to 1.7)	0.12 (Benifuuki vs placebo)
Benifuuki	220.5 ± 28.2	218.8 ± 26.3	−1.8 ± 26.6	0.65	−0.8 (−8.9 to 7.4)	0.85 (Benifuuki vs Yabukita)
Yabukita	217.1 ± 30.3	216.4 ± 31.2	−0.2 ± 20.0	0.94	−5.9 (−14.0 to 2.3)	0.16 (Yabukita vs placebo)
Triglycerides (mg/dl)						
Placebo	122.2 ± 63.5	110.7 ± 56.6	−12.2 ± 53.8	0.12	18.0 (−16.7 to 52.7)	0.31 (Benifuuki vs placebo)
Benifuuki	111.3 ± 46.8	116.6 ± 59.0	5.3 ± 58.2	0.53	−6.4 (−41.1 to 28.3)	0.72 (Benifuuki vs Yabukita)
Yabukita	132.5 ± 86.1	143.7 ± 168.7	11.9 ± 124.0	0.50	24.4 (−9.9 to 58.8)	0.16 (Yabukita vs placebo)
Fasting plasma glucose (mg/dl)						
Placebo	90.0 ± 15.5	88.3 ± 10.2	−1.9 ± 10.8	0.23	0.7 (−4.1 to 5.4)	0.79 (Benifuuki vs placebo)
Benifuuki	86.9 ± 8.5	87.0 ± 9.2	0.1 ± 9.4	0.95	−2.1 (−6.8 to 2.6)	0.39 (Benifuuki vs Yabukita)
Yabukita	87.7 ± 11.1	89.5 ± 19.5	1.8 ± 16.3	0.43	2.7 (−2.0 to 7.4)	0.26 (Yabukita vs placebo)
HbA1c (%)						
Placebo	5.2 ± 0.4	5.2 ± 0.4	0.1 ± 0.2	0.02	−0.01 (−0.1 to 0.0)	0.76 (Benifuuki vs placebo)
Benifuuki	5.1 ± 0.2	5.1 ± 0.3	0.1 ± 0.1	0.01	0.00 (−0.1 to 0.1)	0.10 (Benifuuki vs Yabukita)
Yabukita	5.1 ± 0.3	5.1 ± 0.3	0.1 ± 0.2	0.02	−0.01 (−0.1 to 0.0)	0.76 (Yabukita vs placebo)
Insulin (μU/ml)						
Placebo	10.6 ± 7.4	11.1 ± 8.2	0.5 ± 10.8	0.76	−1.4 (−3.7 to 0.9)	0.22 (Benifuuki vs placebo)
Benifuuki	8.6 ± 2.1	9.5 ± 1.8	0.9 ± 2.7	0.03	−0.6 (−2.8 to 1.7)	0.62 (Benifuuki vs Yabukita)
Yabukita	9.1 ± 2.1	10.1 ± 4.8	1.0 ± 5.0	0.16	−0.9 (−3.1 to 1.4)	0.45 (Yabukita vs placebo)
Serum amyloid A (μg/ml)						
Placebo	16.8 ± 55.5	8.2 ± 1.3	−8.7 ± 56.1	0.28	−0.04 (−2.1 to 2.0)	0.97 (Benifuuki vs placebo)
Benifuuki	14.7 ± 24.2	8.1 ± 0.7	−6.6 ± 24.2	0.06	−1.4 (−3.5 to 0.6)	0.17 (Benifuuki vs Yabukita)
Yabukita	8.5 ± 3.2	9.6 ± 8.6	1.1 ± 9.2	0.43	1.4 (−0.6 to 3.4)	0.18 (Yabukita vs placebo)
High-sensitivity C-reactive protein (mg/dl)						
Placebo	111.1 ± 175.5	88.4 ± 125.2	−20.9 ± 218.7	0.51	−5.1 (−56.2 to 46.0)	0.84 (Benifuuki vs placebo)
Benifuuki	185.5 ± 289.4	89.0 ± 91.3	−96.5 ± 257.3	0.01	−33.7 (−84.9 to 17.6)	0.20 (Benifuuki vs Yabukita)
Yabukita	96.4 ± 152.7	115.1 ± 159.4	17.7 ± 209.2	0.56	28.6 (−21.7 to 78.9)	0.26 (Yabukita vs placebo)
Serum adiponectin (μg/ml)						
Placebo	9.8 ± 4.7	9.8 ± 4.6	0.03 ± 0.1	0.83	0.1 (−0.4 to 0.6)	0.75 (Benifuuki vs placebo)
Benifuuki	10.5 ± 4.5	10.5 ± 3.9	0.04 ± 1.3	0.82	0.3 (−0.2 to 0.8)	0.29 (Benifuuki vs Yabukita)
Yabukita	10.5 ± 4.6	10.2 ± 4.2	−0.2 ± 1.8	0.34	−0.2 (−0.7 to 0.3)	0.46 (Yabukita vs placebo)
8-OHdG concentration (ng/ml)						
Placebo	12.5 ± 7.7	8.5 ± 5.3	−4.0 ± 6.4	< 0.0001	−0.2 (−2.0 to 1.6)	0.82 (Benifuuki vs placebo)
Benifuuki	11.3 ± 7.9	7.9 ± 5.3	−3.5 ± 7.2	0.002	−1.0 (−2.8 to 0.8)	0.27 (Benifuuki vs Yabukita)
Yabukita	12.0 ± 7.1	9.1 ± 5.0	−2.9 ± 7.4	0.009	0.8 (−1.0 to 2.6)	0.39 (Yabukita vs placebo)
8-OHdG/CRE (ng/mg creatinine)						
Placebo	9.2 ± 3.2	7.7 ± 2.4	−1.5 ± 2.9	0.001	−0.3 (−1.0 to 0.4)	0.40 (Benifuuki vs placebo)
Benifuuki	8.8 ± 3.5	7.3 ± 1.8	−1.5 ± 3.0	0.001	−0.4 (−1.1 to 0.3)	0.24 (Benifuuki vs Yabukita)
Yabukita	9.6 ± 3.7	7.9 ± 1.8	−1.7 ± 3.2	0.001	0.1 (−0.6 to 0.8)	0.74 (Yabukita vs placebo)
8-OHdG generation rate (ng/kg/h)						
Placebo	7.6 ± 4.1	6.2 ± 3.4	−1.4 ± 4.0	0.02	0.7 (−0.9 to 2.2)	0.38 (Benifuuki vs placebo)
Benifuuki	7.7 ± 5.4	6.9 ± 5.2	−0.8 ± 6.0	0.34	0.6 (−0.9 to 2.2)	0.42 (Benifuuki vs Yabukita)
Yabukita	8.2 ± 4.1	6.4 ± 3.2	−1.6 ± 4.6	0.02	0.1 (−1.5 to 1.6)	0.95 (Yabukita vs placebo)

^a^Baseline values were compared with those obtained after 12 weeks. The significance of the differences was isolated by the paired *t* test.

^b^The change in the Benifuuki or the Yabukita green tea group minus the change in the placebo group; and the change in the Benifuuki group minus the change in the Yabukita group.

Net differences were calculated by analysis of covariance; adjusted for age (years), sex, and individual baseline variables.

HDL, high-density lipoprotein; HbA1c, glycosylated hemoglobin; 8-OHdG, 8-hydroxy-2’-deoxyguanosine; CRE, creatinine.

## Discussion

In this RCT, we found that LDL-cholesterol levels in the Yabukita green tea group and the Benifuuki green tea group significantly decreased, by 6.9 mg/dl and 9.4 mg/dl, respectively, after intervention for 12 weeks. These results coincide with the meta-analysis of 20 eligible RCTs by Kim et al., which showed that green tea consumption significantly reduced serum LDL-cholesterol by −5.30 mg/l (95% CI −9.99, −0.62 mg/l) [13]. However, no significant differences between the Yabukita green tea group and the Benifuuki green tea group were observed in the present study. Imbe et al. reported that LDL-cholesterol levels decreased by 3.9 mg/dl and 5.0 mg/dl in the Yabukita and Benifuuki green tea groups, respectively, after 12 weeks of intervention [14]. Although the LDL-cholesterol-lowering effect of Benifuuki green tea was greater than that of Yabukita green tea only among subjects without a habit of daily tea drinking, no significant difference was found between the Yabukita and Benifuuki green tea groups when all participants were considered [14].

A previous study showed that EGCG eliminated cholesterol from bile salt micelles by indirectly decreasing the solubility of cholesterol. Kobayashi et al. suggested that EGCG has an inhibitory effect on cholesterol absorption in the intestine through its action on micelles [5]. Suzuki et al. compared the effects of Benifuuki extracts that contain methylated catechins such as EGCG3”Me with those of Yabukita extracts in animal studies of mice fed a high-fat/high-sucrose diet. They found that a 1% Benifuuki diet significantly reduced levels of LDL/very low-density lipoprotein (VLDL)-cholesterol and suppressed the expression of lipogenesis genes [12]. They suggested that the reduction of plasma LDL/VLDL-cholesterol levels might be because the methylated catechins (EGCG3”Me) contained in the Benifuuki suppressed the expression of lipogenesis genes. However, no differences in the LDL-lowering effect between the Benifuuki green tea group and the Yabukita green tea group were observed in the present study. Therefore, further studies on how the presence of EGCG3”Me in green tea contributes to lowering LDL-cholesterol in humans are necessary.

In a previous meta-analysis of 15 RCTs evaluating the effects of green tea catechins on body weight, the included trials were analyzed as three separate pools of data (seven trials evaluated green tea catechins with caffeine compared with a caffeine-matched control group, six trials evaluated green tea catechins with caffeine compared with a caffeine-free control group, and two trials evaluated green tea catechins without caffeine compared with a caffeine-free control group) owing to their heterogeneous nature [15]. The authors concluded that ingestion of green tea catechins with caffeine might affect body weight, but the magnitude of the effect over a median of 12 weeks was small and not likely to be clinically relevant. Green tea catechins with caffeine reduced body weight, but green tea catechins alone did not affect body weight [15]. In the present study, however, changes in body weight were not observed in any of the groups, although participants in the Yabukita green tea group consumed 595.8 mg/day of catechins and 131.6 mg/day of caffeine, and those in the Benifuuki green tea group consumed 629.0 mg/day of catechins and 124.4 mg/day of caffeine for 12 weeks. The reason for this discrepancy may be explained by differences in the participants’ physique. In most previous studies in which a significant reduction in body weight was observed after long-term tea drinking [16–18], the mean BMI of the participants was > 25 kg/m^2^. Since the mean BMI of the participants in the present study was < 25 kg/m^2^, this may explain why we did not observe a reduction in body weight. When evaluating the body-weight lowering effect of green tea, it is important to consider the baseline body weight of the participants as well as the amount of caffeine in the green tea.

In the present study, HDL-cholesterol levels in the Benifuuki green tea group significantly increased, by 2.3 mg/dl (95% CI 0.2, 4.5 mg/dl) compared with those of the placebo group. HDL-cholesterol levels in the Benifuuki green tea group also increased, by 2.7 mg/dl (95% CI 0.6, 4.8 mg/dl) compared with those of the Yabukita green tea group. Some animal model studies reported that green tea extract consumption increased HDL-cholesterol levels and decreased LDL-cholesterol and triglyceride levels; however, the mechanism remains unclear [19,20]. Our results suggest that Benifuuki green tea extract powder has an effect on raising HDL-cholesterol levels in humans.

Our study showed that Yabukita green tea extract powder significantly decreased waist circumference, by 1.5 cm (95% CI −2.7, −0.3 cm) compared with the placebo powder. In a previous study, healthy participants (98 men and 97 women) aged 20–65 years with 22.5 < BMI ≤ 30 kg/m^2^ were assigned to consume either three bottles of placebo drink (control group), or two bottles of catechin-containing drink and one bottle of placebo drink (low-dose group), or three bottles of catechin-containing drink (high-dose group) per day at mealtimes for 12 weeks (daily consumption of catechins was 41.1 mg, 444.3 mg, or 665.9 mg, respectively). That study showed that waist circumference and waist/hip ratio decreased significantly in both catechin groups at 12 weeks (waist: *p* < 0.05 in the low-dose group, *p* < 0.01 in the high-dose group; waist/hip ratio: *p* < 0.01 in both groups) [9]. Although the present results coincide with those of the previous study, this may be by chance or coincidence. The previous study showed that green tea catechins significantly decreased body weight as well as waist circumference; however, we did not observe a reduction in body weight even after 12 weeks of intervention with green tea extract.

This study assessed the lipid-lowering effects of two kinds of green tea in humans. A strength of the study is that our RCT design included a relatively large number of participants and we were able to completely follow up all participants in this study.

There are also some limitations to the study. Because the study participants responded to our invitation in newspapers or flyers, they might have had more interest in their health than the general population of the same generation in that area. This might have caused selection bias.

Furthermore, we examined differences in the lipid-lowering effect of two kinds of green tea, but did not examine the mechanism of the lipid-lowering effect of each component in the green teas. We could not identify which component(s) attributed to the effects observed in this study. Although O-methylated EGCG has been shown to have a lipid-lowering effect in mice, it seems unlikely that the lipid-lowering effect of green tea (catechins) is attributed to O-methylated EGCG in humans. We did not collect any information on dietary patterns from our participants and could not evaluate the effects on the obtained results. However, the randomization procedure means that the dietary pattern may not differ between the groups.

Recently, green tea consumption in Japan has decreased, especially in younger generations, and the increase in CVD among young people has become a public health concern [21]. Under these circumstances, it is important to elucidate the relationship between the kinds of tea that Japanese people consume on a regular basis and the risks of CVD, in order to provide a simple and effective method of preventing CVD.

The results of this study may be generalizable to other areas. Elucidation of the influence of specific green tea components on artery-related indices will contribute to the restraint of metabolic syndrome and help to reduce the risk of CVDs such as stroke. Therefore, this information will also contribute towards maintaining the health of older generations in Japan’s aging society. Furthermore, elucidation of catechins’ mechanism of improving the lipid profile of the blood may lead to the development of novel drugs for dyslipidemia. It is hoped that elucidation of the lipid-lowering effects of green tea will contribute towards the prophylaxis of lifestyle-related diseases.

In conclusion, Yabukita green tea and Benifuuki green tea lowered LDL-cholesterol, and the lipid-lowering effects of these two green teas were not different. Neither kind of green tea lowered body weight.
